# Response of Myeloid Leukemia Cells to Luteolin is Modulated by Differentially Expressed Pituitary Tumor-Transforming Gene 1 (PTTG1) Oncoprotein

**DOI:** 10.3390/ijms19041173

**Published:** 2018-04-12

**Authors:** Pei-Yi Chen, Hsin-Jung Tien, Shih-Fen Chen, Chi-Ting Horng, Huei-Lin Tang, Hui-Ling Jung, Ming-Jiuan Wu, Jui-Hung Yen

**Affiliations:** 1 Center of Medical Genetics, Buddhist Tzu Chi General Hospital, Hualien 970, Taiwan; pyc571@gmail.com; 2 Department of Molecular Biology and Human Genetics, Tzu Chi University, Hualien 970, Taiwan; tiencelia@gmail.com (H.-J.T.); damaruco.csf@gmail.com (S.-F.C.); sophiatang0205@gms.tcu.edu.tw (H.-L.T.); 3 Department of Ophthalmology, Kaohsiung Armed Force General Hospital, Kaohsiung 804, Taiwan; h56041@gmail.com; 4 Department of Pharmacy, Kaohsiung Armed Force General Hospital, Kaohsiung 804, Taiwan; tracy530917@gmail.com; 5 Department of Biotechnology, Chia Nan University of Pharmacy and Science, Tainan 717, Taiwan; mingjiuanwu@gmail.com

**Keywords:** luteolin, PTTG1, myeloid leukemia cells, apoptosis, cell proliferation

## Abstract

Luteolin, a flavonoid nutraceutical abundant in vegetables and fruits, exhibits a wide range of bioactive properties, including antioxidant, anti-inflammatory and anti-cancer activities. Pituitary tumor-transforming gene 1 (PTTG1), an oncoprotein that regulates cell proliferation, is highly expressed in several types of cancer cells including leukemia. In this study, we aim to investigate the anti-cancer effects of luteolin on cells with differential PTTG1 expression and their underlying mechanisms in human myeloid leukemia cells. Methyl thiazolyl tetrazolium (MTT) assay data showed that luteolin (25–100 μM) significantly reduced cell viability in THP-1, HL-60 and K562 cells but did not affect normal peripheral blood mononuclear cells (PBMCs). Flow cytometric analysis and Western blot data demonstrated that luteolin induced a stronger apoptosis on undifferentiated myeloid leukemia cells with higher PTTG1 protein levels than on 12-myristate 13-acetate (PMA)- or all-trans-retinoic acid (ATRA)-differentiated cells with lower PTTG1 expression. Furthermore, PTTG1 knockdown by shRNA in leukemia cells suppressed cell proliferation, arrested cell-cycle progression and impaired the effectiveness of luteolin on cell-cycle regulation. Moreover, PTTG1-knockdown cells with luteolin exposure presented a reduction of the apoptotic proteins and maintained higher levels of the anti-apoptotic proteins such as Mcl-1, Bcl-2 and p21, which exhibited greater resistance to apoptosis. Finally, microarray analysis showed that 20 genes associated with cell proliferation, such as *CXCL10*, *VEGFA*, *TNF*, *TP63* and *FGFR1*, were dramatically down-regulated in PTTG1-knockdown cells. Our current findings clearly demonstrate that luteolin-triggered leukemic cell apoptosis is modulated by the differential expression of the PTTG1. PTTG1 oncoprotein overexpression may modulate cell proliferation-related regulators and enhance the response of myeloid leukemia cells to luteolin. Luteolin is beneficial for the treatment of cancer cells with highly expressed PTTG1 oncoprotein.

## 1. Introduction

Human myeloid leukemia is a highly heterogeneous neoplasm characterized by genetic mutations that enhance the proliferation of myeloid blasts, promote their self-renewal, and/or block hematopoietic differentiation. Recurrent chromosomal aberrations and molecular markers are well established for the diagnosis and prognosis of acute or chronic myeloid leukemia (AML or CML) [1,2]. However, the current treatment of patients with leukemia still yields poor outcomes, with expected cure rates in the order of 30–40%, depending on the biological characteristics of the leukemic clone. Drug resistance and severe side effects of chemotherapeutic agents reduce the clinical efficacy of leukemia therapy [3,4]. Thus, there is an urgent need to develop alternative regimens or synergistic drugs with low toxicity and high specificity for the improved efficacy of current leukemia therapy. Dietary phytochemicals are found to be an important resource to develop effective anti-cancer agents [5]. Many studies have found that these natural agents successfully interrupt DNA replication, arrest cell-cycle progression, and trigger apoptosis of leukemia cells but do not affect normal or quiescent blood cells [6,7,8,9].

Pituitary tumor-transforming gene 1 (PTTG1), also known as securin, is a protein that possesses separase inhibitor activity and regulates the cell cycle during the mitotic phase in normal tissue. The degradation of PTTG1 protein by anaphase-promoting complex (APC/C) at the metaphase–anaphase transition allows separase to fulfill its function in sister-chromatid separation during the cell cycle. The PTTG1 protein is also known to serve as a cell-cycle modulator in regulating both G1/S and G2/M phase transitions. PTTG1 functions in cell replication, DNA damage/repair, metabolism and transactivation activity [10,11,12]. PTTG1 is a transcription factor, and overexpressed PTTG1 protein acts as an oncogenic protein for promoting cell-cycle progression, cell proliferation and tumorigenesis [13,14]. The PTTG1 protein is abundantly expressed in various invasive tumors and hematopoietic malignancies; however, its level is low or undetectable in most normal tissues [13,15]. Highly expressed PTTG1 is also correlated with metastasis, invasiveness and poor prognosis in several types of tumors [16,17,18,19,20]. The mechanisms by which PTTG1 promotes tumorigenesis seem to be diverse and include causes of chromosome aberrations, the regulation of apoptosis, the modulation of DNA damage response, and the induction of angiogenesis [21]. Several genes, including *c-Myc*, p53, *FGF2*, p21, prolactin and *MMP2*, have been reported to be the transcriptional targets of PTTG1 in cell proliferation and cell cycle regulation [22]. Our previous study showed that PTTG1 expression is dramatically attenuated via increases in KLF6, a tumor suppressor, during PMA-induced myeloid cell differentiation [23]. In addition to the modulation of tumorigenesis, differential PTTG1 expression is reported to affect the responses to chemoprevention and chemotherapy. Anti-cancer agents such as oxaliplatin modulate the cytotoxicity induction of cancer cells via regulation of PTTG1 protein expression in human colorectal cancer cells [24]. These findings indicated that the PTTG1 protein may modulate cell-cycle progression and cell differentiation, maintain the precursor stages of cancer cells, and regulate the chemotherapeutic effects of anti-cancer agents.

**Figure 1:**
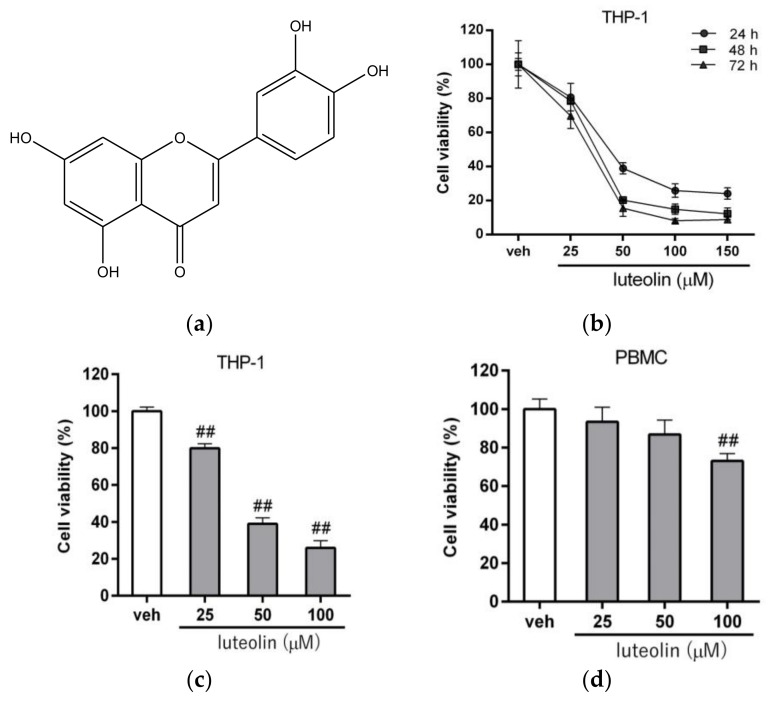
Effects of luteolin on the viability of human myeloid leukemia cells. (**a**) Chemical structure of luteolin. (**b**) THP-1 cells were treated with vehicle (0.1% dimethyl sulfoxide (DMSO)) or luteolin (25, 50, 100 and 150 μM) for 24 h, 48 h and 72 h, respectively. Cell viability was measured by MTT assay. The data are presented as the means ± standard deviation (SD) from three independent experiments. THP-1 cells (**c**) and peripheral blood mononuclear cells (PBMCs) (**d**) were treated with vehicle or luteolin (25, 50 and 100 μM) for 24 h, and cell viability was determined by MTT assay. The data are presented as the means ± SD from three independent experiments. ## *p* < 0.01 represents a significant difference compared to the vehicle-treated cells (veh).

Luteolin (3′,4′,5,7-tetrahydroxyflavone, Figure 1a), a flavonoid nutraceutical found in many vegetables and fruits, exhibits a wide range of bioactive properties, including antioxidant, anti-inflammatory, antimicrobial, neuroprotection and anti-cancer activities [25,26,27,28]. Several studies have confirmed that luteolin inhibits cell proliferation and induces cell-cycle arrest and apoptosis in a variety of cancer cells, including lung cancer, colon cancer, prostate cancer, breast cancer, nasopharyngeal carcinoma and hepatocellular carcinoma cells [29,30,31,32,33,34]. In human leukemia studies, luteolin has been reported to inhibit cell growth and induce apoptosis in human myeloid leukemia HL-60 cells [35,36]. Luteolin possesses selective inhibitory activity against Fms-like tyrosine kinase 3 (FLT3), a receptor tyrosine kinase highly expressed in AML patients, and induces a strong cytotoxic effect in MV4-11 leukemia cells [37]. Luteolin also significantly induced apoptosis in chronic lymphocytic leukemia (CLL) cell lines by increasing the caspase activities and triggering the intrinsic apoptotic pathway [38]. One recent study showed that luteolin targets p90 ribosomal S6 kinase (RSK) to suppress cell growth in MOLM-13 and Kasumi-1 leukemic cells [39]. These studies suggested that luteolin is an important chemo-preventive or chemotherapeutic agent that induces cancer cell apoptosis in leukemia.

Luteolin has been reported to mediate apoptosis via both the intrinsic and extrinsic apoptosis pathways [40]. Luteolin can activate caspase 3 or 9 and modulate anti-apoptotic proteins such as Bcl-2 family members for the induction of cancer cell apoptosis in vitro and in vivo [36,41,42,43,44]. Previous studies have demonstrated that the molecular targets of luteolin involved in the apoptotic process include p21, p53 and Bcl-2 [41]. These above findings suggested that luteolin is a potent anti-cancer agent that functions by inducing the apoptosis of leukemia cells. Differential expression of the PTTG1 protein is known to regulate cancer cell progression and the chemotherapeutic effects of anti-cancer agents. However, the anti-cancer effectiveness of luteolin in cancer cells with differentially expressed PTTG1 remains unclear. In the present study, we aim to investigate the effects of PTTG1 expression on luteolin-mediated anti-cancer activity and their underlying mechanisms in human myeloid leukemia cells. Our study provides new insight into the chemotherapeutic effects of luteolin on hematopoietic malignancies.

## 2. Results

### 2.1. Luteolin Reduced the Viability of Human Myeloid Leukemia Cells

To verify the anti-leukemic effect of luteolin, we first examined the cytotoxic effect of luteolin on human acute myeloid leukemia THP-1 cells. The THP-1 cells were treated with luteolin (25–150 μM) for 24–72 h, and the cell viability was measured by MTT assay. The viability of luteolin-treated cells was significantly reduced in a dose- and time-dependent manner (Figure 1b). As shown in Figure 1c, the viability of cells treated with luteolin (25, 50 and 100 μM) for 24 h significantly decreased from 100.0 ± 2.3% to 79.9 ± 2.4%, 38.9 ± 3.3% and 25.9 ± 4.0%, respectively, compared to the vehicle-treated group (*p* < 0.01). The IC_50_ value in THP-1 cells was determined to be 46.16 μM. It has been reported that that PTTG1 expression in normal PBMC was very low or undetectable [13,23]. Therefore, we further analyzed the effect of luteolin on PBMCs. The viability of PBMCs treated with luteolin (25–100 μM) was higher than that of luteolin-treated leukemia cell groups, with values from 100.0 ± 5.3% to 93.4 ± 7.5%, 86.8 ± 7.2% and 73.2 ± 3.7%, respectively (Figure 1d). Similar effects were also found in human myeloid leukemia HL-60 and K562 cell lines treated with luteolin. The viability of luteolin (25–100 μM)-treated HL-60 and K562 cells also markedly decreased from 100.0 ± 4.4% to 38.0 ± 2.1%, 14.2 ± 1.6% and 20.0 ± 3.7% and from 100.0 ± 4.0% to 69.5 ± 7.3%, 38.1 ± 7.8% and 26.2 ± 2.7%, respectively (*p* < 0.01) (Figure S1). The IC_50_ values in HL-60 and K562 cells were determined to be 16.14 μM and 41.16 μM, respectively. These data suggested that luteolin exhibited differential anti-cancer effects on distinct types of myeloid leukemia cells. The leukemia cells were more responsive to luteolin than normal PBMCs.

### 2.2. Effects of Luteolin on the Viability of Undifferentiated and Differentiated Leukemia Cells with Differential Pituitary Tumor-Transforming Gene 1 (PTTG1) Expression

**Figure 2:**
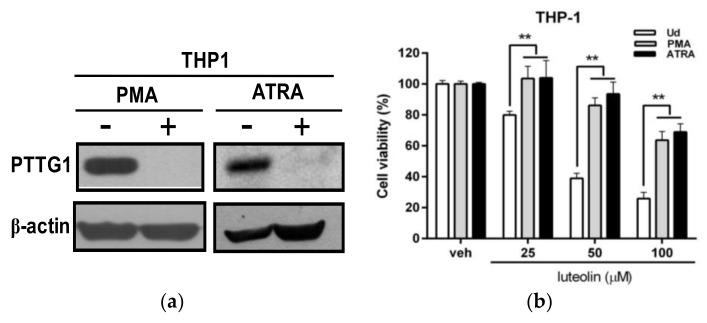
Effects of luteolin on the viability of THP-1 cells with differential pituitary tumor-transforming gene 1 (PTTG1) expression. (**a**) THP-1 cells were pretreated with PMA (200 nM) or ATRA (10 μM) for 72 h. The levels of PTTG1 and β-actin protein were determined by Western blot analysis. The experiments were performed at three times, and a representative blot is shown. (**b**) THP-1 cells were pretreated with PMA (200 nM) or ATRA (10 μM) for 72 h differentiation, and then, differentiated THP-1 cells were incubated with vehicle or luteolin (25, 50 and 100 μM) for 24 h. Cell viability was determined by MTT assay. ** *p* < 0.01 represents a significant difference compared to the PMA- or ATRA-untreated cells (Ud group).

It is known that differentiating agents such as phorbol 12-myristate 13-acetate (PMA) and all-trans-retinoic acid (ATRA) drive myeloid leukemia cells, such as THP-1, HL-60 or K562 cells, toward differentiation and a normal cell-like phenotype. We previously demonstrated that PTTG1 is not expressed in normal PBMCs and that PTTG1 expression is significantly reduced in PMA-differentiated THP-1 cell lines [23]. To investigate the cytotoxic effect of luteolin in myeloid leukemia cells with differential PTTG1 expression, we first determined the PTTG1 protein level in PMA- and ATRA-differentiated THP-1 cells. The cells were pretreated with PMA (200 nM) or ATRA (10 uM) for 72 h to induce cell differentiation, and the PTTG1 protein level was measured by Western blot analysis. As shown in Figure 2a, the PTTG1 protein level was dramatically attenuated in PMA- and ATRA-differentiated THP-1 cells. We further examined the cytotoxic effect of luteolin in differentiated THP-1 cells. The differentiated cells were incubated with luteolin (25–100 μM) for 24 h, and the viability of undifferentiated THP-1 cells was more significantly decreased by luteolin than that of PMA- or ATRA-differentiated cells at the same concentration of luteolin treatment (*p* < 0.01) (Figure 2b). The PTTG1 protein level was also significantly reduced in PMA- or ATRA-induced differentiated HL-60 and K562 cells (Figure S2a,c). Cell viability was decreased in luteolin-treated undifferentiated HL-60 and K562 cells compared with the PMA- or ATRA-differentiated cells (Figure S2b,d). These data indicated that the undifferentiated leukemia cells with a high level of PTTG1 protein are more sensitive to luteolin-mediated cytotoxicity.

### 2.3. Luteolin Induced More Significant Cell Apoptosis in Undifferentiated Myeloid Leukemia Cells with Abundant PTTG1 Protein

**Figure 3:**
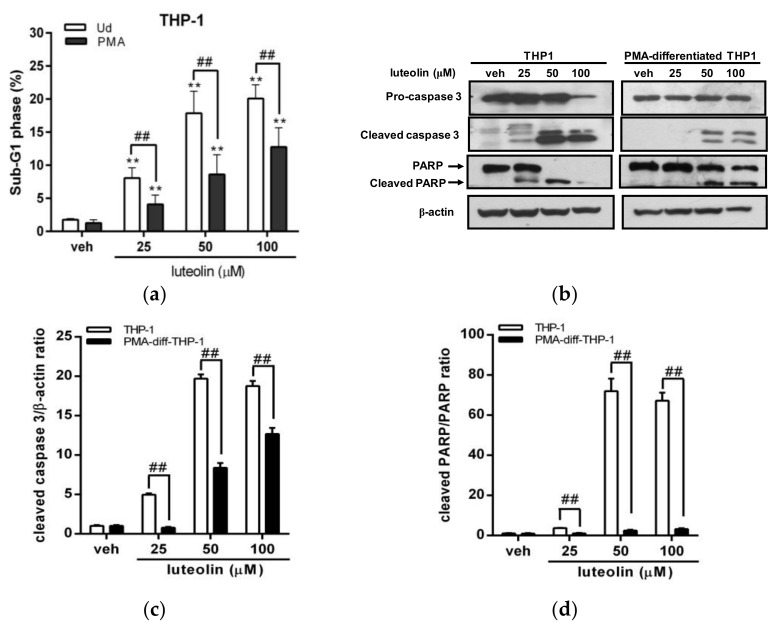
Effects of luteolin on the apoptosis of THP-1 cells with differential PTTG1 expression. (**a**) The undifferentiated or PMA-differentiated THP-1 cells were treated with vehicle or luteolin (25–100 μM) for 24 h, and then, the cell population in sub-G1 phase was determined by flow cytometric analysis. The experiments were triplicated, and the data are presented as the means ± SD from three independent experiments. ** *p* < 0.01 represents a significant difference compared to the vehicle group (veh). ## *p* < 0.01 represents a significant difference compared to the undifferentiated cells (Ud group). (**b**) Apoptotic marker proteins, including pro-caspase 3, cleaved caspase 3, PARP1 and cleaved PARP1, were analyzed by Western blot analysis. The experiments were triplicated, and a representative blot is shown. (**c**) The intensity of cleaved caspase 3 versus β-actin proteins is normalized and presented as the mean ± SD of triplicated experiments. (**d**) The intensity of cleaved PARP1 versus total PARP1 proteins is normalized and presented as the mean ± SD of triplicated experiments. ## *p* < 0.01 represents a significant difference compared to the undifferentiated THP-1 cells (THP-1 group).

To investigate the effect of cell apoptosis induced by luteolin in cells with differential expression of PTTG1, we further analyzed the cell apoptotic effect of luteolin on undifferentiated or PMA-differentiated THP-1 cells using flow cytometric analysis. As shown in Figure 3a, the percentage of the cell population in sub-G1 phase (dead cell population) in luteolin-treated THP-1 cells (25, 50 and 100 μM) was significantly increased from 1.8% to 8.1%, 17.9% and 20.1%, respectively, compared with the vehicle group (*p* < 0.01). However, treatment of PMA-differentiated THP-1 cells with luteolin (25–100 μM) significantly attenuated the cell population in the sub-G1 phase to approximately 4.1%, 8.6% and 12.8%, respectively, compared to undifferentiated cell groups (1.3%) (*p* < 0.01). We further analyzed apoptosis-related markers, including cleaved caspase 3 and poly (ADP-ribose) polymerase 1 (PARP1), using Western blot analysis. As shown in Figure 3b–d, we found that treatment of undifferentiated THP-1 cells with luteolin markedly increased the levels of apoptotic protein markers, including cleaved caspase 3 and PARP1 proteins. In contrast, the cleaved forms of caspase 3 and PARP1 proteins were not or were slightly detected in PMA-differentiated THP-1 cells under the same conditions. These results indicated that luteolin induced less severe apoptosis in human myeloid leukemia cells that are differentiated by PMA, which have less PTTG1 protein.

**Figure 4:**
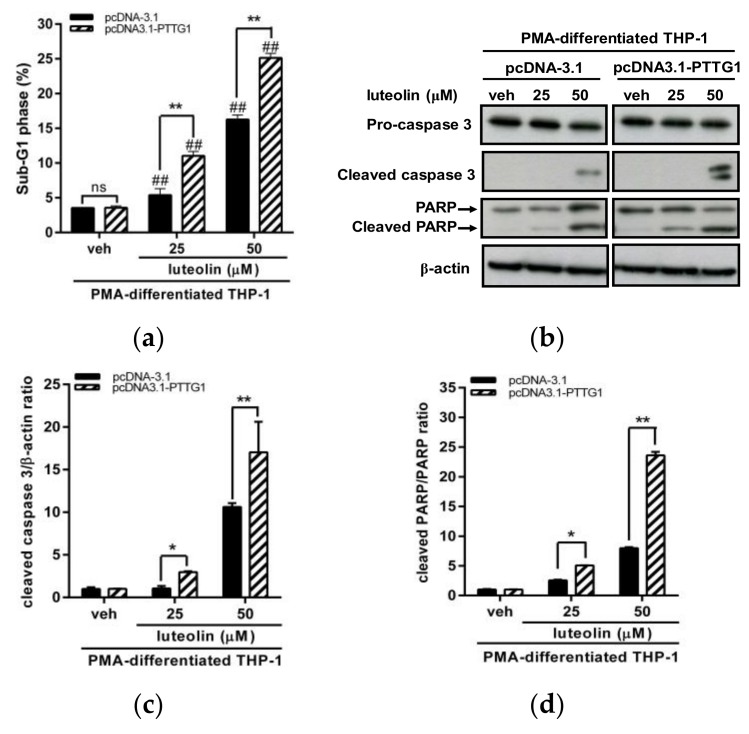
Effects of PTTG1 overexpression on luteolin-induced apoptosis in PMA-differentiated THP-1 cells. (**a**) The PMA-differentiated THP-1 cells were transfected with pcDNA-3.1 control vector or pcDNA3.1-PTTG1 expression plasmid for 48 h followed by treatment of vehicle or luteolin (25 and 50 μM) for 24 h, and then, the cell population in sub-G1 phase of cell cycle was determined by flow cytometric analysis. The experiments were triplicated, and the data are presented as the means ± SD from three independent experiments. ## *p* < 0.01 represents a significant difference compared to vehicle-treated cells. ** *p* < 0.01 represents a significant difference compared to the pcDNA-3.1 control vector-transfected cells. “ns” represents no significant. (**b**) Proteins including pro-caspase 3, cleaved caspase 3, PARP1, cleaved PARP1 and β-actin were analyzed by Western blot analysis. The experiments were triplicated, and a representative blot is shown. (**c**) The intensity of cleaved caspase 3 versus β-actin proteins is normalized and presented as the mean ± SD of triplicated experiments. (**d**) The intensity of cleaved PARP1 versus total PARP1 proteins is normalized and presented as the mean ± SD of triplicated experiments. * *p* < 0.05 and ** *p* < 0.01 represent a significant difference compared to the pcDNA-3.1 control vector-transfected cells.

Furthermore, we investigated whether differentiated cells with enhanced PTTG1 were associated with an increased sensitivity for luteolin. We evaluated the effects of overexpression of PTTG1 on the cell cycle and cell apoptosis after luteolin treatment. The PMA-differentiated THP-1 cells were transfected with pcDNA-3.1 vector or pcDNA3.1-PTTG1 expression plasmid followed by treatment of the vehicle or luteolin. First, we examined the effect of luteolin on the cell-cycle distribution in these transfected cells. The results of the cell-cycle analysis are shown in Figure S3. In vehicle-treated cells, overexpression of PTTG1 could not affect the cell-cycle distribution in G1, S and G2/M phases (Figure S3a–c). After luteolin treatment, enhanced PTTG1 expression did not significantly alter the cell population in the G1 phase (Figure S3a). However, increased PTTG1 expression followed by luteolin treatment promoted cell-cycle arrest in the S phase (Figure S3b) and decreased cell population in G2/M phase (Figure S3c). These data suggested that PTTG1 overexpression in differentiated THP-1 cells may modulate the effect of luteolin on cell-cycle distribution. Next, we investigated the effect of PTTG1 overexpression on the luteolin-induced apoptosis. PTTG1 overexpression in PMA-differentiated cells followed by luteolin treatment significantly increased the cell death population in sub-G1 phase (Figure 4a) and markedly elevated the levels of cleaved caspase 3 and PARP1 proteins (Figure 4b–d). These data indicated that differentiated cells with enhanced PTTG1 are more sensitive to luteolin. These above results suggested that PTTG1 expression is crucial for the response of luteolin in human AML cells.

### 2.4. Effects of PTTG1 Knockdown on Cell Growth and the Luteolin Response in Human Myeloid Leukemia Cells

**Figure 5:**
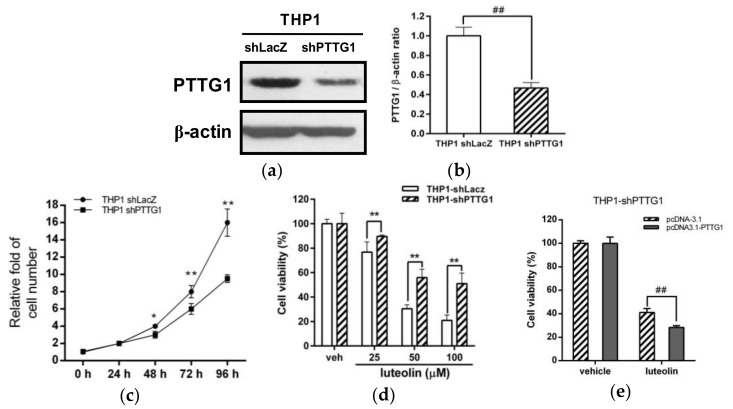
Effects of PTTG1 knockdown on cell proliferation and luteolin-induced cell death in THP-1 cells. (**a**) Control LacZ- and PTTG1-knockdown THP-1 cells (THP1 shLacZ and THP1 shPTTG1) were generated as described in the Materials and Methods. The PTTG1 and β-actin protein levels were determined by Western blot analysis. The experiments were triplicated, and a representative blot is shown. (**b**) The intensity of PTTG1 versus β-actin is normalized and presented as the mean ± SD of triplicated experiments. ## *p* < 0.01 represents a significant difference compared to the shLacZ control cells (THP1 shLacZ group). (**c**) Cells were stained with trypan blue, and cell numbers were measured by counting viable cells at the 0-, 24-, 48-, 72- and 96 h time points, respectively. The experiments were replicated at three times. * *p* < 0.05 and ** *p* < 0.01 represent a significant difference compared to the THP1 shLacZ group. (**d**) The shLacZ and shPTTG1 cells were treated with vehicle or luteolin (25–100 μM) for 24 h, and cell viability was determined by MTT assay. The experiments were triplicated. The data are presented as the means ± SD of three independent experiments. ** *p* < 0.01 represents a significant difference compared to the THP1 shLacZ control group. (**e**) The pcDNA-3.1 control vector or pcDNA3.1-PTTG1 expression plasmid was transfected to THP1 shPTTG1 cells for 24 h followed by treatment of luteolin (100 μM) for 24 h. Cell viability was determined by MTT assay. ## *p* < 0.01 represents a significant difference compared to the pcDNA-3.1 control-transfected cells.

The above findings suggested that higher expression of PTTG1 in undifferentiated leukemia cells were associated with a higher apoptotic response to luteolin administration. To elucidate this hypothesis, we generated PTTG1-knockdown cells by shRNA in leukemia cell lines. We successfully generated a PTTG1-knockdown THP-1 cell clone (THP1 shPTTG1), and the PTTG1 protein level was dramatically reduced by approximately 54% compared with that in shLacZ control cells (Figure 5a,b). As shown in Figure 5c, cell proliferation was significantly inhibited in PTTG1-knockdown cells compared to THP1 shLacZ control cells. Furthermore, we investigated the effect of luteolin on the viability of PTTG1-knockdown cells. As shown in Figure 5d, the viability of PTTG1 knockdown cells treated with luteolin (25–100 μM) was higher than that of luteolin-treated cells in the shLacZ control group (*p* < 0.01). These effects of PTTG1-knockdown on cell proliferation and luteolin response were confirmed in another PTTG1-knockdown cell clone (THP1 shPTTG1#16) (Figure S4). A similar effect on cell viability was also found in luteolin-treated HL-60- or K562-shPTTG1 cells (Figure S5). Moreover, to investigate whether rescue of PTTG1 protein expression altered the luteolin-induced cell death in the PTTG1-knockdown cells, the THP1 shPTTG1 cells were transfected with pcDNA3.1-PTTG1 expression plasmid followed by treatment of luteolin (100 μM) and cell viability was measured using MTT assay. As shown in Figure 5e, the rescue of the PTTG1 expression significantly reduced the cell viability of luteolin-treated cells. These data verified that PTTG1 knockdown in leukemia cells caused the attenuation of cell proliferation and resistance to luteolin-mediated cell death.

### 2.5. Effects of Luteolin on Cell-Cycle Distribution and Apoptosis in PTTG1-Knockdown Cells

**Figure 6:**
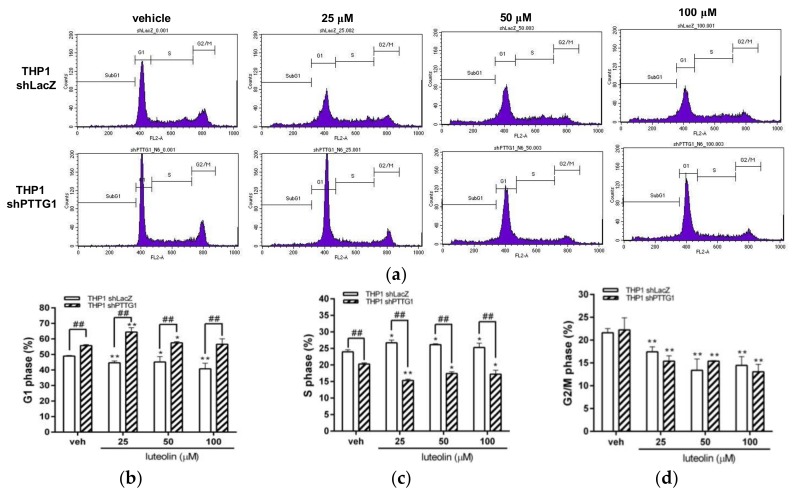
Effects of luteolin on cell-cycle distribution in PTTG1-knockdown cells. The THP1 shLacZ and shPTTG1 cells were treated with vehicle or luteolin (25–100 μM) for 24 h. (**a**) The cell-cycle distribution was determined by flow cytometric analysis. The experiments were replicated three times. A representative histogram is shown. Cell populations in the (**b**) G1 phase (**c**) S phase (**d**) G2/M phase were quantified. The experiments were replicated at three times. The data are presented as the means ± SD from three independent experiments. * *p* < 0.05 and ** *p* < 0.01 indicate a significant difference compared to the vehicle group (veh). ## *p* < 0.01 represents a significant difference compared to the THP1 shLacZ control cells.

To further elucidate the effects of luteolin on the cell cycle and apoptosis in PTTG1-knockdown cells, THP1 shLacZ and THP1 shPTTG1 cells were treated with luteolin (25–100 μM) for 24 h, followed by flow cytometric analysis. The results of the cell-cycle analysis are shown in Figure 6a. In luteolin-treated THP1 shLacZ cells, the cell population in the S phase was increased (Figure 6c); however, the cell populations in the G1 and G2/M phases were decreased (Figure 6b,d). These data indicated that luteolin induced S phase arrest in THP-1 leukemia cells. In PTTG1-knockdown cells, cells treated with or without luteolin presented significantly increased cell populations in the G1 phase (Figure 6c). The cell populations in the S and G2/M phases were decreased in luteolin-treated cells (Figure 6b,d). These data suggested that PTTG1 knockdown in THP-1 cells leads to cell-cycle arrest in the G1 phase and modulates the effect of luteolin on cell-cycle progression.

**Figure 7:**
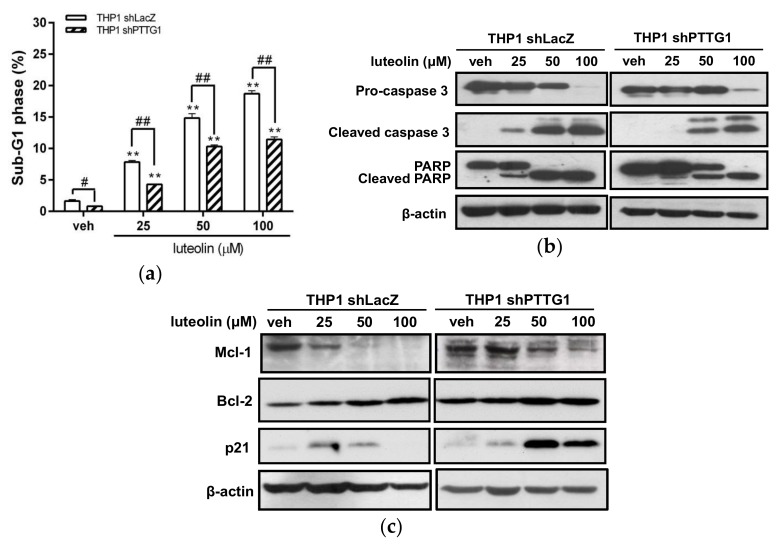
Effects of luteolin on the apoptosis of PTTG1-knockdown THP-1 cells. The THP1 shLacZ and shPTTG1 cells were treated with vehicle or luteolin (25–100 μM) for 24 h. (**a**) Cell population in the sub-G1 phase was determined by flow cytometric analysis. The experiments were triplicated, and the data are presented as the means ± SD from three independent experiments. ** *p* < 0.01 represents a significant difference compared to the vehicle group (veh). # *p* < 0.05 and ## *p* < 0.01 represent a significant difference compared to the THP1 shLacZ control cells. (**b**) The protein expression levels of pro-caspase 3, cleaved caspase 3, PARP1, cleaved PARP1 and β-actin proteins were analyzed by Western blot analysis. The experiments were triplicated, and a representative blot is shown. (**c**) The Mcl-1, Bcl-2, p21 and β-actin proteins were analyzed by Western blot analysis. The experiments were triplicated, and a representative blot is shown.

We further assessed the apoptotic effect of luteolin on PTTG1-knockdown THP-1 cells. The dead cell population (sub-G1 phase) was significantly reduced in THP1 shPTTG1 cells with luteolin treatment compared with that in shLacZ control cells (Figure 7a). As shown in Figure 7b, THP1 shLacZ cells treated with 25 μM luteolin displayed increased levels of cleaved caspase 3 and PARP1, while the shPTTG1 cells were not affected under the same conditions. Moreover, at higher concentrations of luteolin (50–100 μM) treatment, PTTG1-knockdown cells displayed strongly decreased cleavage of both caspase 3 and PARP1 compared with luteolin-treated shLacZ control cells. These data verified that PTTG1 protein expression potentiated luteolin-mediated cell apoptosis.

We further examined the effect of luteolin on anti-apoptotic proteins, such as myeloid cell leukemia sequence 1 (Mcl-1), Bcl-2 and p21, in PTTG1-knockdown cells. As shown in Figure 7c, PTTG1 knockdown maintained higher levels of Mcl-1, Bcl-2 and p21 proteins in luteolin-treated THP1 shPTTG1 cells than in THP1 shLacZ control cells. These data indicated that elevating the levels of anti-apoptotic proteins in leukemia cells with a lower amount of PTTG1 may result in greater resistance to luteolin-mediated cell death.

### 2.6. Gene Expression Alterations in PTTG1-Knockdown Leukemia Cells

To elucidate the molecules involved in the PTTG1-potentiated anti-leukemia effect of luteolin on myeloid leukemia cells, microarray analysis was performed to determine differentially expressed genes in THP1 shLacZ control and THP1 shPTTG1 cells. The genes with relative transcription levels (*p* < 0.05) with log_2_(ratio) ≥ 1.0 were up-regulated, and those with log_2_(ratio) ≤ −1.0 were down-regulated. A total of 493 differentially expressed genes were detected in THP1 shPTTG1 cells compared with the control cells (THP1 shLacZ cells). Among these genes, 116 gene transcripts were up-regulated, and 377 genes were down-regulated. The top 5 up-regulated and down-regulated genes were listed in Tables S1 and S2. We further validated the mRNA expression of down-regulated genes including *CXCL10* and *BCL3* by quantitative reverse-transcription polymerase chain reaction (Q-RT-PCR) analysis and the data is shown in Figure S6. According to gene set enrichment analysis, the 493 differentially expressed genes were grouped into different categories based on biological process Gene Ontology (GO) terms. Twenty genes associated with the positive regulation of cell proliferation process category were significantly down-regulated in PTTG1-knockdown cells and listed in Table 1. Among these genes, 15 genes, namely, *CXCL10*, *VEGFA*, *TNF*, *TP63*, *FGFR1*, *CCL2*, *STAT1*, *CDK6*, *KIT*, *PTPRC*, *JUN*, *CD40*, *VEGFC*, *PRAME* and *KRAS* have been reported to be associated with the regulation of cancer cell proliferation in hematological malignancies. The top 5 differentially expressed genes (*CXCL10*, *VEGFA*, *TNF*, *TP63* and *FGFR1*) were decreased to approximately 0.10-, 0.15-, 0.20-, 0.24- and 0.30-fold in PTTG1-knockdown THP-1 cells compared to THP1 shLacZ control cells, respectively. Down-regulation of PTTG1 gene expression was associated with a reduction in the levels of gene transcripts for cell proliferation. These data suggested that PTTG1 overexpression promotes leukemia cell proliferation and enhances luteolin-mediated leukemia cell apoptosis by modulating positive regulators of cell proliferation.

## 3. Discussion

The current treatment of patients with myeloid leukemia still faces problems of drug resistance and the severe side effects of chemotherapeutic agents. The development of nutraceuticals such as flavonoids from dietary sources to inhibit cancer cell progression is a safe and feasible approach. Luteolin is a potent anti-cancer nutraceutical that prevents cancer development by inactivating several signals and transcriptional pathways essential for cancer cell proliferation. Despite extensive studies of the effects of luteolin on chemoprevention, the exact mechanisms or molecular targets of luteolin through which it affects leukemia progression remain unclear. In this study, we demonstrated that luteolin induced more cytotoxicity in human acute myeloid leukemia (AML) cells, such as THP-1 and HL-60 cells, and in human chronic myeloid leukemia (CML) K562 cells, than in normal PBMCs. In a previous study, it has been demonstrated that the levels of PTTG1 protein were different in these cell lines (HL-60 > K562 > THP-1) [23]. In this study, the IC_50_ values of luteolin treatment in HL-60, K562 and THP-1 were 16.14 μM, 41.16 μM and 46.16 μM, respectively (HL-60 < K562 < THP-1). These findings suggested that the higher level of PTTG1 expression in these myeloid leukemia cells was correlated with the lower IC_50_ value of luteolin. Luteolin has been reported to inhibit cancer cell proliferation mainly through arresting cell-cycle progression. Luteolin kills cancer cells by promoting the cell apoptotic program in several types of cancer [44]. In this study, we demonstrated that luteolin induced cell-cycle arrest in the S1 phase in THP-1 leukemia cells. Luteolin increased the cell population in the sub-G1 phase and the levels of apoptotic protein markers, including cleaved caspase 3 and PARP1, in THP-1 leukemia cells. Caspase 3 is a critical component of the cell apoptotic process due to its location in the protease cascade downstream. Our results showed that luteolin induces apoptosis in leukemia cell lines by increasing the cleavage of caspase 3. These results revealed that luteolin suppressed cell proliferation by inhibiting cell-cycle progression and activated the apoptotic pathways to induce cancer cell death in human myeloid leukemia cells. Our current findings indicated that luteolin may serve as a chemo-preventive agent against human hematological malignancies such as AML or CML.

It has been reported that leukemia cells can be differentiated by treatment with agents such as PMA or ATRA and exhibit normal phenotypic characteristics, and that these differentiated cells showed more resistant to cytotoxic or apoptotic effects than their parent cells [7,8,45]. These different effects may depend on the regulatory molecules and distinct cellular status. In hematological malignancies, PTTG1 oncoprotein is overexpressed and has been reported to promote cell proliferation, regulate the cell cycle, and modulate apoptosis in cancer cells [46,47]. However, the PTTG1 protein is dramatically down-regulated in normal PBMCs and PMA-differentiated leukemia cells [23]. In the present study, we hypothesized that the anti-leukemic effects of luteolin may be modulated by the PTTG1 protein and are associated with its expression level. We found that the PTTG1 mRNA expression was proportionally reduced in the THP-1 cells treated with PMA (200 nM) for 24–72 h. The cell death induced by luteolin (25–100 μM) in 24 h- or 72 h-PMA-differentiated THP-1 cells was also proportionally inhibited. We further confirmed that the level of PTTG1 protein was very low in the PMA- or ATRA-differentiated THP-1, HL-60, and K562 cells. Additionally, we demonstrated that PBMC is resistance to luteolin. These data supported the idea that normal PBMC or differentiated cells with lower or undetectable PTTG1 levels were associated with higher cell viability under luteolin treatment conditions. In this study, we showed that luteolin is less toxic to normal PBMC or differentiated cells. Since the role of PTTG1 on human normal CD34 (+) hematopoietic stem cells (HSC) is unclear, we detected the expression level of PTTG1 in human CD34 (+) hematopoietic stem cells (HSC). We found that the levels of PTTG1 mRNA expression in THP-1, PMA-differentiated THP-1, PBMC and CD34(+) HSC were THP-1 >> PMA-differentiated THP-1 ≈ CD34(+) HSC > PBMC. This data indicated that the level of PTTG1 expression in CD34(+) HSC was similar to that of PMA-differentiated cells. Therefore, we suppose that the hematopoietic stem cells are resistant to luteolin-mediated cytotoxicity.

Furthermore, we demonstrated that luteolin increased the death cell population in sub-G1 phase and the activation of apoptotic proteins in undifferentiated THP-1 leukemia cells compared with corresponding PMA-differentiated cells. Moreover, our data demonstrated that the PMA-differentiated AML cells with enhanced PTTG1 expression could modulate the effect of luteolin on cell-cycle progression. We found that overexpression of PTTG1 in PMA-differentiated cells were associated with increases of luteolin-induced cell apoptosis. Our findings indicated that the PTTG1 protein plays a critical role in the different responses to luteolin treatment in undifferentiated and differentiated leukemia cells. Here, we provide the first evidence that the anti-cancer effects of luteolin are modulated by PTTG1 levels in human myeloid leukemia cells. Several studies demonstrated that PTTG1 was also highly expressed in lymphocytic leukemia cell lines such as Jurkat and MOLT-4 cells. Moreover, PTTG1 overexpression was detected in many histological subtypes of lymphoma [13,14,48]. These findings suggested that the involvement of PTTG1 oncoprotein in lymphoid neoplasms. Whether the effect of luteolin on lymphocytic leukemia cells is associated with PTTG1 expression remains unclear and needs to be further investigated.

PTTG1 has been reported to regulate the effectiveness of anti-cancer drugs in some cancer cell lines [49,50]. PTTG1 overexpression induced an increase in mitotic cells and apoptosis in paclitaxel-treated prostate cancer cell lines [51]. Higher levels of PTTG1 expression may enhance the apoptotic effects of the anti-microtubule drug BPR0L075 on human colorectal cancer cells [50]. To clarify the role of PTTG1 in the anti-leukemic effects of luteolin, we further generated PTTG1-knockdown leukemia cells in this experiment. In the present study, PTTG1 knockdown in THP-1 cells resulted in inhibition of cell proliferation and cell-cycle arrest in the G1 phase. PTTG1 knockdown decreased the cleavage of both caspase 3 and PARP1, which indicated that PTTG1 expression levels modulated the apoptotic effect induced by luteolin in human myeloid leukemia cells. Our findings revealed that high levels of PTTG1 protein expression in leukemia will promote cell-cycle progression. In leukemia cells with down-regulated PTTG1 expression, the cells did not pass the G1/S checkpoint, and the cell cycle was arrested in the G1 phase. Luteolin targets the cell cycle machinery and induces proliferating leukemia cells to arrest in the S phase, promoting apoptosis. Thus, the unproliferating PTTG1-knockdown cells showed greater resistance to the effects of luteolin.

Targeting the apoptotic pathways is a promising therapeutic strategy in human myeloid leukemia. Activation of apoptosis machinery by interacting anti-apoptotic proteins, such as Bcl-2 family members, can kill leukemia cells. The anti-apoptotic Bcl-2 family members are significantly down-regulated by various apoptotic stimuli [52]. In the present study, we found that the knockdown of PTTG1 in THP-1 cells increased the levels of the anti-apoptotic proteins Bcl-2 and Mcl-1 compared to the levels in THP1 control cells. When cells were treated with luteolin, PTTG1-knockdown THP-1 cells maintained higher levels of anti-apoptotic proteins than shLacZ control cells. These data indicated that increases in the levels of anti-apoptotic proteins in PTTG1-knockdown cells could contribute to greater resistance to luteolin-induced apoptosis. It has been demonstrated that luteolin promotes apoptosis partly by decreasing the levels of Bcl-x(L), Mcl-1, p21 (CIP1/WAF) and Mdm-2 [30]. Cheng et al. reported that luteolin induced cancer cell apoptosis via cleavage of Bcl-2 and Bcl-X_L_ in human leukemia HL-60 cells [36]. The Bcl-2 is a critical determinant of the overall predisposition of a cell to undergo apoptosis. Up-regulation of the anti-apoptotic factor Bcl-2 so supports the absence of apoptosis in Pttg^-/-^ pituitary glands [53]. Mcl-1 is a key member of the Bcl-2 anti-apoptotic family, is commonly overexpressed in a number of cancers, and is a major cause of resistance to radio- and chemotherapies [54]. These results suggested that up-regulation of Bcl-2 family members in PTTG1-knockdown cells may increase cell resistance to luteolin-mediated apoptosis.

p21 protein was first recognized as a cyclin-dependent kinase inhibitor (CKI) and is considered one of the key regulators of the cell cycle and apoptosis [55]. Inactivation or depletion of p21 sensitizes cells to apoptosis following DNA damage [56]. In contrast, ectopic expression of p21 delays apoptosis induced by several kinds of DNA damage in various cell lines and in a mouse model [57]. PTTG1 has been reported to bind the p21 promoter and inhibit its transcriptional activity [53]. Consistent with these findings, massive levels of p21 induction were evident in shPTTG1 cells at high doses of luteolin (50 and 100 μM) treatment. As p21 functions as a mediator of cell-cycle arrest and an inhibitor of apoptosis, PTTG1 likely suppresses p21 to promote drug-induced apoptosis. Our current results also suggested that the presence of PTTG1 enhanced luteolin-induced cell death via inhibition of p21 and that PTTG1 knockdown decreased the transcriptional suppression that causes p21 induction and cell resistance to apoptosis.

Our current findings revealed that PTTG1 overexpression may enhance the effectiveness of luteolin by regulating the molecules involved in cell proliferation and cell-cycle progression in human myeloid leukemia cells. In this study, microarray analysis was carried to elucidate the molecules involved in the PTTG1-potentiated anti-cancer effect of luteolin on leukemia cells. Twenty genes associated with positive regulation of the cell proliferation process were significantly down-regulated in PTTG1-knockdown THP-1 cells. Among these down-regulated genes in PTTG1-knockdown cells, the top 5 differentially expressed genes were *CXCL10*, *VEGFA*, *TNF*, *TP63* and *FGFR1*. These five genes have been reported to be involved in cell proliferation in leukemia cells. The chemokine ligand 10 (CXCL10) mRNA was significantly reduced in PTTG1-knockdown cells. Recently, CXCL10 was reported to have higher expression levels in CML patients and may be involved in cancer cell growth and metastasis [58]. It has been reported that VEGF/VEGF receptor (VEGFR) expression is up-regulated in hematolymphoid tumor cells accompanied by angiogenesis [59]. TNF-α is involved in cancer cell proliferation and leukemogenesis [60]. The TP63 gene, a member of the TP53 family of transcription factors, has been reported to play a critical role in tumor development in a variety of human cancers, including hematological malignancies [61,62]. Additionally, increases in the level of TP63 may increase the chemo-sensitivity of human cancer cells to anti-cancer drugs, such as doxorubicin and cisplatin [63,64,65]. These data indicated that high expression levels of p63 correlated with poor prognosis in hematological malignancies. It will be interesting to investigate whether PTTG1 directly targets the p63 promoter and how PTTG1 interacts with p63 in the process of cancer drug treatment. Hematologic malignancies are associated with FGFR1 abnormalities present in acute myeloid leukemia (AML), lymphoblastic leukemia/lymphoma and mixed phenotype acute leukemia [66]. Our findings indicated that PTTG1 overexpression triggers the expression of several genes promoting cell proliferation and cell-cycle progression in human myeloid leukemia cells. Whether high levels of PTTG1 expression directly affects these cell-proliferative regulators to sensitize the apoptotic response to luteolin in human myeloid leukemia cells remains unknown and needs to be further investigated.

The development of apoptosis-triggering nutraceuticals is a promising approach in therapies for leukemia patients. Our current results have clearly demonstrated that luteolin-triggered leukemic cell apoptosis might be modulated by the differential expression of the PTTG1 oncoprotein. Our current findings provide a novel mechanism for the chemotherapeutic action of luteolin in various cancer cells with PTTG1 overexpression. In this study, we investigated the in vitro effect of luteolin on myeloid leukemia cells. Recently, it has been reported that NSGS mice are good models and provide optimal conditions for engraftment and expansion of human leukemia cells in vivo [67,68,69,70]. To test the effect of luteolin on leukemia in vivo, these human AML cell lines can be used to generate a xenograft model to further examine the anti-leukemic effect of luteolin in NSGS mice.

## 4. Materials and Methods

### 4.1. Materials and Chemicals

Luteolin was purchased from Tokyo Chemical Industry (Tokyo, Japan). Dimethyl sulfoxide (DMSO), phorbol 12-myristate 13-acetate (PMA), all-trans-retinoic acid (ATRA), 3-(4,5-dimethylthiazol-2-yl)-2,5-diphenyl tetrazolium bromide (MTT), propidium iodide (PI), RPMI-1640 medium, nonessential amino acids (NEAA) and other chemicals were purchased from Sigma-Aldrich Co. (St. Louis, MO, USA) unless otherwise indicated.

### 4.2. Cell Culture

The THP-1 (human acute monocytic leukemia), HL-60 (human acute promyelocytic leukemia) and K562 (chronic myelogenous leukemia) cell lines were obtained from Bioresource Collection and Research Center (Hsinchu, Taiwan). Peripheral blood mononuclear cells (PBMCs) from one healthy volunteer donor were isolated using Ficoll-Paque Plus Reagent (GE Healthcare, Buckinghamshire, UK). The cells were maintained in RPMI-1640 medium (2 mM glutamine, 1.5 g/L sodium bicarbonate, 4.5 g/L glucose, 10 mM HEPES and 1 mM sodium pyruvate) supplemented with 10% fetal bovine serum (FBS) (Thermo Fisher Scientific, Inc., Rockford, IL, USA) and 1% NEAA in a 5% CO_2_ incubator at 37 °C. For cell differentiation, the cells were seeded onto 6-well plates and treated with PMA (200 nM) or ATRA (10 μM for THP-1 and HL-60 cells or 8 μM for K562 cells) for 72 h.

### 4.3. Cell Viability

Cell viability was measured using MTT assay. Cells were treated with the vehicle (0.1% DMSO) or luteolin (25–100 μM) for 24 h followed by incubation with 1 mg/mL MTT solution at 37 °C for 3 h. The cell pellets were collected by centrifugation, and the crystals were dissolved in DMSO. The extent of the reduction of MTT by mitochondria was determined as the absorbance at 550 nm. For determining the viable cell number of THP1 shLacZ or THP1 shPTTG1 cells, cells were stained with trypan blue and cell numbers were measured by counting viable cells at the 0-, 24-, 48-, 72- and 96 h time points.

### 4.4. Flow Cytometric Analysis

Cells were seeded on 6-well plate and treated with vehicle or luteolin (25–100 μM) for 24 h. For cell-cycle analysis, the cells were harvested, washed with ice-cold phosphate-buffered saline (PBS) and fixed with 70% (*v*/*v*) ethanol at −20 °C for 24 h. The cells were then washed with ice-cold PBS and incubated in propidium iodide (PI) staining buffer (20 μg/mL propidium iodide, 200 μg/mL RNase A and 0.1% (*v*/*v*) Triton X-100 in PBS) in the dark at room temperature for 30 min. Cell-cycle analysis was carried out on a FACSCalibur, and cell distribution was analyzed by Cell Quest Pro software (BD Biosciences, San Jose, CA, USA) to determine the population of cells in sub-G1, G1, S and G2/M phases.

### 4.5. Western Blot Analysis

Total cellular lysates were extracted using RIPA buffer (Thermo Fisher Scientific, Rockford, IL, USA). The proteins were separated by 10% sodium dodecyl sulfate polyacrylamide gel electrophoresis (SDS-PAGE), transferred onto a polyvinylidene difluoride (PVDF) membrane (PerkinElmer, Boston, MA, USA) and incubated with the following specific antibodies: anti-caspase 3, anti-PARP, anti-Mcl-1, and anti-Bcl-2 (GeneTex, Irvine, CA, USA); anti-cleaved caspase 3 and anti-p21Waf1/Cip1 (Cell Signaling Technology, Danvers, MA, USA); and anti-PTTG1 (Thermo Fisher Scientific, Rockford, IL, USA) and anti-β-actin (Sigma-Aldrich, St. Louis, MO, USA). The immunoblots were incubated with the appropriate HRP-conjugated secondary antibodies (Santa Cruz Biotechnology, Santa Cruz, CA, USA) and were detected using Amersham ECL™ Prime Western Blotting Detection Reagent. The signal was visualized on Amersham Hyperfilm™ ECL (GE Healthcare, Buckinghamshire, UK).

### 4.6. Generation of PTTG1-Knockdown Stable Cell Clones

The control LacZ- and PTTG1-knockdown THP-1, HL-60 and K562 cells were established by lentiviruses encoding specific shRNA obtained from the National RNAi Core Facility (Academia Sinica, Taipei, Taiwan) (LacZ-shRNA clone: TRCN0000072233; PTTG1-shRNA clone: TRCN0000013711). These knockdown clones were selected with puromycin (2 μg/mL) and isolated for a single and pure stable cell clone. The expression level of PTTG1 protein in the PTTG1-knockdown clones was characterized by Western blot analysis.

### 4.7. Transfection of PTTG1 Expression Plasmids

The PTTG1 expression plasmid pcDNA3.1-PTTG1 [20] was kindly provided by Dr. Ji-Hshiung Chen (Tzu Chi University, Hualien, Taiwan). The THP1 shPTTG1 cells or PMA-differentiated cells were transfected with pcDNA3.1 vector or pcDNA3.1-PTTG1 plasmid using TransIT^®^-2020 Trasnfection reagent (Mirus Bio, Madison, WI, USA) or Lipofectamine reagent (Thermo Fisher Scientific, Rockford, IL, USA) according to the manufacturer’s instruction. After 24 or 48 h transfection, cells were treated with vehicle or luteolin for 24 h and further analysis.

### 4.8. RNA Preparation and Microarray Analysis

Total RNA was extracted from THP1 shLacZ and THP1 shPTTG1 cells using TRIzol reagent according to the manufacturer’s instructions (Thermo Fisher Scientific, Rockford, IL, USA). RNA quality and concentration were determined with OD_260_/OD_280_ ≥ 1.8 and OD_260_/OD_230_ ≥ 1.5 for acceptable purity. The integrity of RNA samples was measured using an Agilent RNA 6000 Nano Assay (Agilent Technology, Inc., Santa Clara, CA, USA), and an RNA integrity number (RIN) value > 6 was required for further microarray analysis. The RNA samples (1 μg) for the generation of amino allyl antisense RNA (aa-aRNA) was performed using an Amino Allyl MessageAmp II aRNA Amplification Kit (Ambion, CA, USA). The Cy5-labeled aRNAs were prepared for microarray hybridization to the Human Whole Genome One Array^®^ Version 6.1 (HOA 6.1, Phalanx Biotech Group, Hsinchu, Taiwan). The fluorescence intensities of each spot were analyzed by GenePix 4.1 (Molecular Devices, Sunnyvale, CA, USA). The normalized spot intensities were transformed to log_2_ ratios of gene expression between the THP1 shLacZ and THP1 shPTTG1 cell groups. The differentially expressed genes are established at log_2_(ratio) ≥ 1.0 or ≤−1.0 and *p*-value < 0.05 for selection criteria and further analysis.

### 4.9. Quantitative Reverse-Transcription Polymerase Chain Reaction (Q-RT-PCR) Analysis

The RNA was extracted from cells using the Total RNA Mini Kit (Geneaid, Taipei, Taiwan). Reverse transcription was carried out using the High-Capacity cDNA Reverse Transcription Kit (Thermo Fisher Scientific, Rockford, IL, USA). Quantitative real-time PCR was performed by a reaction mixture containing cDNA, human-specific primers [ PTTG1, 5′-aaagctctgttcctgcctca-3′ (forward) and 5′-gagaggcactccactcaagg-3′ (reverse); CXCL10, 5′-aggaacctccagtctcagca-3′ (forward) and 5′-caaaattggcttgcaggaat-3′ (reverse); BCL3, 5′-ccctataccccatgatgtgc-3′ (forward) and 5′-ggtgtctgccgtaggttgtt-3′ (reverse); GAPDH, 5′-atgagaagtatgacaacagcct-3′ (forward) and 5′-agtccttccacgataccaaagt-3′ (reverse)] and the Maxima SYBR Green/ROX qPCR Master Mix (Thermo Fisher Scientific, Rockford, IL, USA). Real-time PCR amplification was performed with a Roche LightCycler^®^-480 Real-Time PCR System. The ∆∆*C*t method was used to measure the relative differences in mRNA expression and normalize with the GAPDH mRNA level in the same samples.

### 4.10. Statistical Analysis

All experiments were repeated at least three times, and the values are expressed as the mean ± SD. The results were analyzed using one-way analysis of variance (ANOVA) with Dunnett’s post hoc test, and a *p*-value < 0.05 was considered statistically significant.

## 5. Conclusions

Luteolin (25~100 µM) possesses a therapeutic potential to inhibit cell growth and trigger the apoptosis of myeloid leukemia cells but does not significantly affect normal PBMCs. Luteolin displayed a stronger apoptosis on undifferentiated myeloid leukemia cells with higher PTTG1 expression levels than on PMA- or ATRA-differentiated cells with lower PTTG1 expression levels. PTTG1 knockdown suppressed cell proliferation, arrested cell-cycle progression and exhibited greater resistance to luteolin-mediated apoptosis. The reduction of PTTG1 expression down-regulated cell proliferation-related genes, impaired the effect of luteolin on cell-cycle regulation and maintained higher levels of anti-apoptotic proteins. High expression levels of PTTG1 may enhance the response of myeloid leukemia cells to luteolin, which may be beneficial for the anti-leukemic effects of luteolin.

## Figures and Tables

**Table 1:** Genes associated with the positive regulation of cell-proliferation process categories based on biological process Gene Ontology terms that are down-regulated in PTTG1-knockdown cells.

Gene Symbol	Description	Log_2_ (shPTTG1/shLacZ)
*CXCL10*	chemokine (C-X-C motif) ligand 10	−3.39
*VEGFA*	vascular endothelial growth factor A	−2.74
*TNF*	tumor necrosis factor	−2.33
*TP63*	tumor protein p63	−2.05
*FGFR1*	fibroblast growth factor receptor 1	−1.78
*CCL2*	chemokine (C-C motif) ligand 2	−1.56
*F3*	coagulation factor III	−1.47
*STAT1*	signal transducer and activator of transcription 1 91 kDa	−1.40
*IL12A*	interleukin 12A	−1.38
*CDK6*	cyclin-dependent kinase 6	−1.35
*KIT*	v-kit Hardy-Zuckerman 4 feline sarcoma viral oncogene homolog	−1.27
*PTPRC*	protein tyrosine phosphatase receptor type C	−1.27
*JUN*	jun proto-oncogene	−1.24
*CD40*	CD40 molecule TNF receptor superfamily member 5	−1.24
*VEGFC*	vascular endothelial growth factor C	−1.23
*PRAME*	preferentially expressed antigen in melanoma	−1.21
*IL12RB2*	interleukin 12 receptor beta 2	−1.16
*VASH2*	vasohibin 2	−1.13
*TGFBR2*	transforming growth factor beta receptor II (70/80 kDa)	−1.09
*KRAS*	v-Ki-ras2 Kirsten rat sarcoma viral oncogene homolog	−1.02

## Supplementary Materials

Supplementary materials can be found at http://www.mdpi.com/1422-0067/19/4/1173/s1. The top 5 up-regulated genes upon knockdown of PTTG1 in THP-1 cells, Table S2: The top 5 down-regulated genes upon knockdown of PTTG1 in THP-1 cells, Figure S1: Effects of luteolin on the viability of leukemia HL-60 and K562 cells, Figure S2: Effects of luteolin on the viability of HL-60 and K562 cells with differential PTTG1 expression, Figure S3: Effects of PTTG1 overexpression on cell-cycle distribution in luteolin-treated differentiated THP-1 cells, Figure S4: Effects of PTTG1-knockdown on cell proliferation and luteolin-induced cell death in cell clone THP1 shPTTG1#16, Figure S5: Effects of PTTG1 knockdown on luteolin-induced cell death in HL-60 and K562 cells, Figure S6: Validation of the down-regulated genes on PTTG1-knockdown THP-1 cells.
