# Supplementary material for: Response of Myeloid Leukemia Cells to Luteolin is Modulated by Differentially Expressed Pituitary Tumor-Transforming Gene 1 (PTTG1) Oncoprotein
Source: Int J Mol Sci. 2018 Apr 12;19(4):1173. doi: 10.3390/ijms19041173 (PMC5979486; doi:10.3390/ijms19041173)
Supplement: Supplementary file 1 [file ijms-19-01173-s001.pdf]

Supplemental Tables and Figures

Table S1. The top 5 up-regulated genes upon knockdown of PTTG1 in THP-1 cells

Gene Symbol	Description	Log_2 (shPTTG1/shLacZ)
KLB	klotho beta	6.60
SEZ6L	seizure related 6 homolog (mouse)-like	4.17
PLAZG2E	phospholipase A2, group IIE	2.50
LTC4S	leukotriene C4 synthase	2.02
CDA	cytidine deaminase	2.01

Table S2. The top 5 down-regulated genes upon knockdown of PTTG1 in THP-1 cells

Gene Symbol	Description	Log_2 (shPTTG1/shLacZ)
CD248	endosialin	-4.92
IFI27	Interferon alpha-inducible protein 27	-4.44
BEX1	brain expressed X-linked 1	-3.81
CXCL10	chemokine (C-X-C motif) ligand 10	-3.39
BCL3	B-cell CLL/lymphoma 3	-3.44

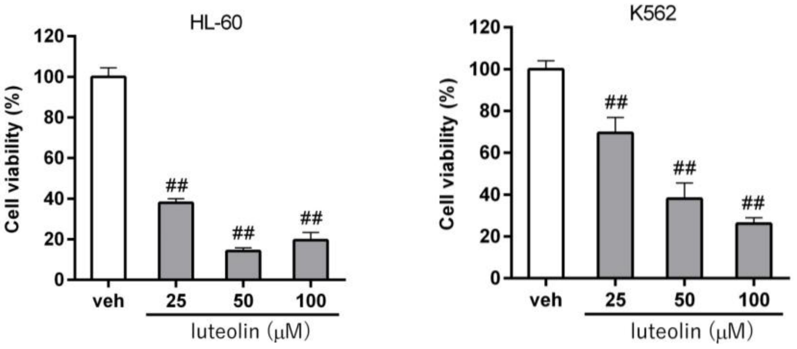

(a) (b)

Figure S1. Effects of luteolin on the viability of leukemia HL-60 and K562 cells. (a) HL-60 cells (b) K562 cells were treated with vehicle or luteolin (25, 50 and 100 μM) for 24 h, and cell viability was determined by MTT assay. The data are presented as the means ± SD from three independent experiments. ##p<0.01 represents a significant difference compared to the vehicle-treated cells (veh).

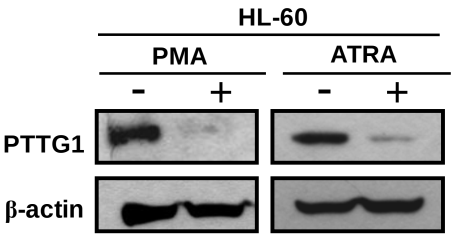

(a)

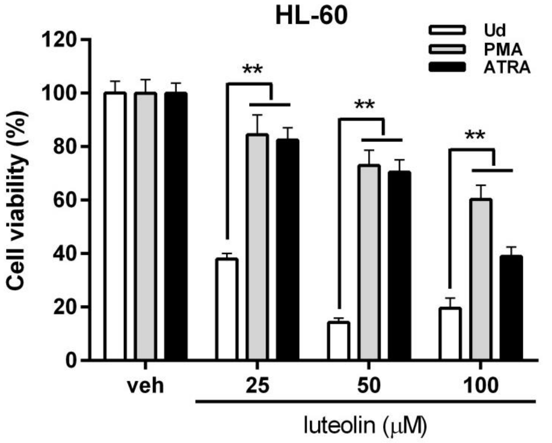

(b)

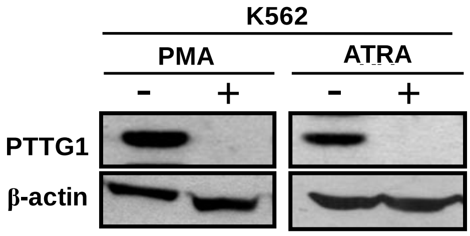

(c)

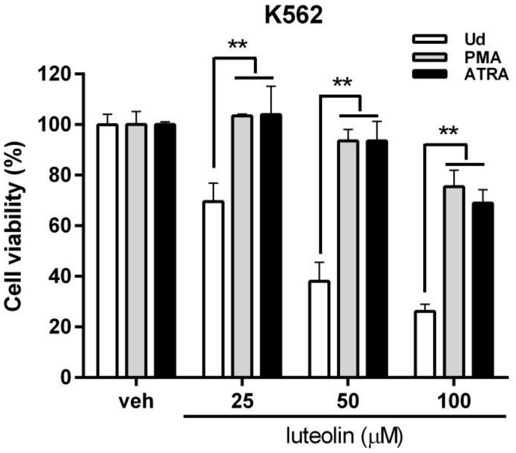

(d)

Figure S2. Effects of luteolin on the viability of HL-60 and K562 cells with differential PTTG1 expression. HL-60 and K562 cells were pretreated with PMA or ATRA for 72 h. The levels of PTTG1 and β-actin proteins in undifferentiated or differentiated (a) HL-60 cells (c) K562 cells were determined by Western blot analysis. The experiments were performed at three times, and a representative blot is shown. The undifferentiated (Ud) and PMA- or ATRA-differentiated (b) HL-60 cells (d) K562 cells were incubated with vehicle or luteolin (25, 50 and 100 μM) for 24 h. Cell viability was determined by MTT assay. ***p*<0.01 represents a significant difference compared to the PMA- or ATAR-untreated cells (Ud group).

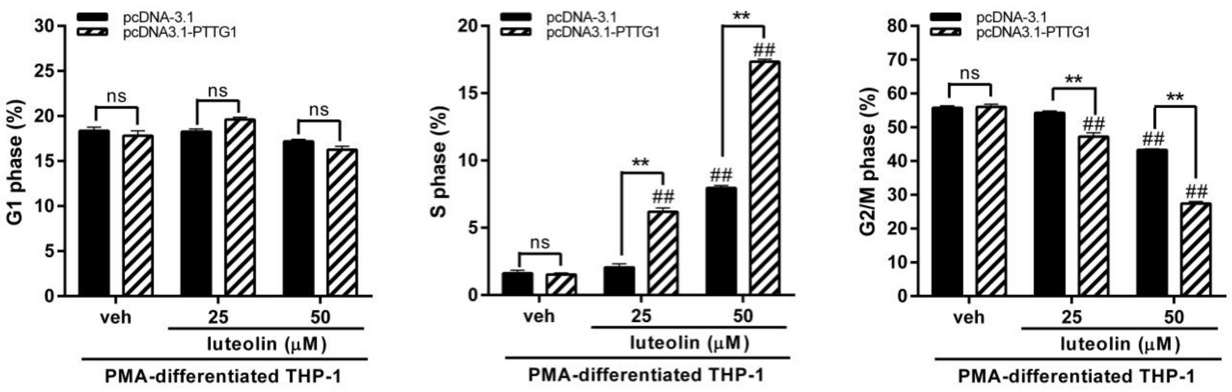

(a) (b) (c)

Figure S3. Effects of PTTG1 overexpression on cell cycle distribution in luteolin-treated differentiated THP-1 cells. The PMA-differentiated THP-1 cells were transfected with pcDNA-3.1 control vector or pcDNA3.1-PTTG1 expression plasmid for 48 h followed by treatment of vehicle or luteolin (25 and 50 μM) for 24 h, and then, the cell population in cell cycle was determined by flow cytometric analysis. Cell populations in (a) G1 phase (b) S phase (c) G2/M phase were quantified. The experiments were replicated at three times. The data are presented as the means ± SD from three independent experiments. ##*p*<0.01 indicates a significant difference compared to the vehicle group (veh). ***p*<0.01represents a significant difference compared to the pcDNA-3.1 control vector-transfected cells. "ns" represents no significant.

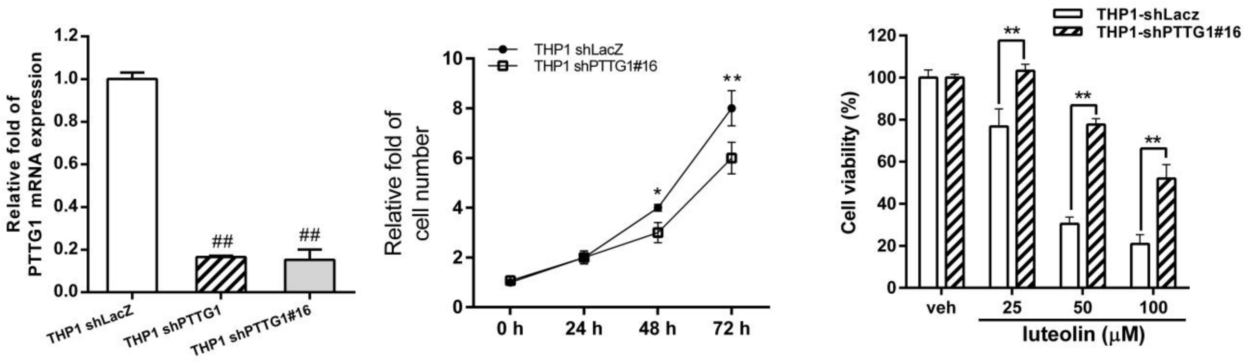

(a) (b) (c)

Figure S4. Effects of PTTG1-knockdown on cell proliferation and luteolin-induced cell death in cell clone THP1 shPTTG1#16. (a) PTTG1 mRNA levels of THP1 shLacZ, THP1 shPTTG1 and THP1 shPTTG1#16 cells were determined by Q-RT-PCR analysis. The experiments were triplicated. The data are presented as the means ± SD of three independent experiments. ##$p<0.01$ represents a significant difference compared to the THP1 shLacZ group. (b) Cells were stained with trypan blue, and cell numbers were measured by counting viable cells at the 0-, 24-, 48- and 72 h time points, respectively. The experiments were replicated at three times. (c) The shLacZ and shPTTG1#16 cells were treated with vehicle or luteolin (25-100 μM) for 24 h, and cell viability was determined by MTT assay. The experiments were triplicated. The data are presented as the means ± SD of three independent experiments. *$p<0.05$ and **$p<0.01$ represent a significant difference compared to the THP1 shLacZ control group.

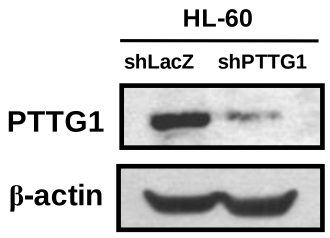

(a)

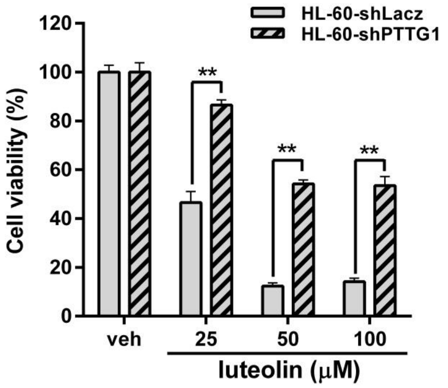

(b)

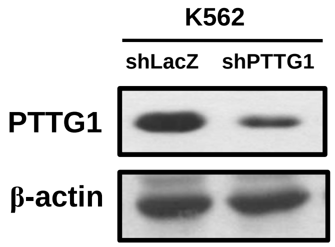

(c)

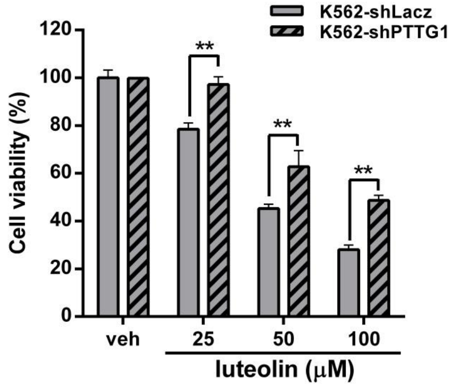

(d)

Figure S5. Effects of PTTG1 knockdown on luteolin-induced cell death in HL-60 and K562 cells. The PTTG1 and β-actin protein levels in (a) HL-60-shLacZ and HL-60-shPTTG1 (c) K562-shLacZ and K562-shPTTG1were determined by Western blot analysis. The experiments were triplicated, and a representative blot is shown. The (b) HL-60-shLacZ and HL-60-shPTTG1 (d) K562-shLacZ and K562-shPTTG1 cells were treated with vehicle or luteolin (25-100 μM) for 24 h, and cell viability was determined by MTT assay. The experiments were triplicated. The data are presented as the means ± SD of three independent experiments. **p<0.01 represents a significant difference compared to the shLacZ control group.

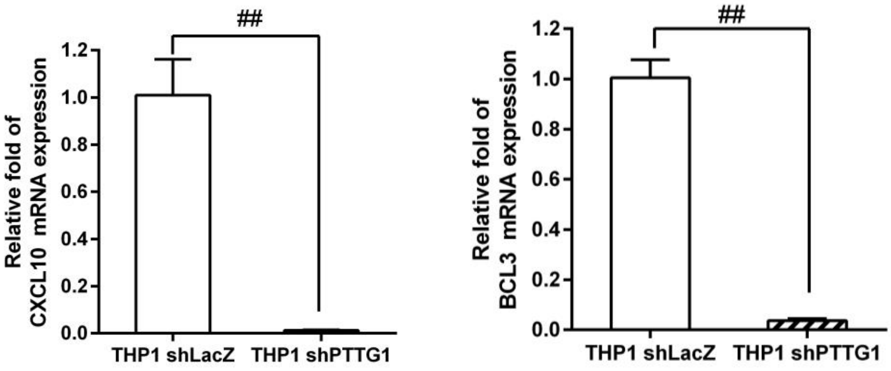

(a) (b)

Figure S6. Validation of the down-regulated genes on PTTG1-knockdown THP-1 cells. The levels of (a) CXCL10 and (b) BCL3 mRNA were determined by Q-RT-PCR analysis. The experiments were triplicated. The data are presented as the means ± SD of three independent experiments. ##p<0.01 represents a significant difference compared to the THP1 shLacZ group.
